# Endoscopic transnasal anterior release and posterior reduction without odontoidectomy for irreducible atlantoaxial dislocation

**DOI:** 10.1186/s13018-019-1167-0

**Published:** 2019-05-06

**Authors:** Xiangsheng Tang, Xinjie Wu, Mingsheng Tan, Ping Yi, Feng Yang, Qingying Hao

**Affiliations:** 1 Department of Spinal Surgery, China-Japan Friendship Hospital, Beijing, 100029 People’s Republic of China; 2 Graduate School of Peking Union Medical College, Beijing, 100730 People’s Republic of China

**Keywords:** Atlantoaxial, Reduction, Anterior release, Endoscopy, Transnasal

## Abstract

**Background:**

To investigate the efficacy and safety of endoscopic transnasal anterior release and posterior reduction without odontoidectomy to treat irreducible atlantoaxial dislocation (IAAD).

**Methods:**

A series of 9 patients with IAAD underwent endoscopic transnasal anterior release and posterior reduction without odontoidectomy. Etiology, instrumentation, fusion rate, and complications were documented. All patients were assessed clinically and radiologically for neurological recovery using the Japanese Orthopedic Association (JOA) score, atlantodontoid interval (ADI), and cervicomedullary angle (CMA).

**Results:**

The mean age of the patients was 41.6 years, ranging from 14 to 60 years. Pathology showed os odontoideum in 3 patients, old traumatic dens fracture in 3 patients, occipitalization of C1 in 2 patients, and rheumatoid arthritis in 1 patient. Seven patients underwent C1–C2 pedicle screw fixations, and 2 patients required occipitocervical fixation. Eight cases resulted in complete reduction and 1 in partial reduction. Complications included one superficial infection related to the posterior approach. All patients were followed up for an average of 17 (range 13–32) months. Bony fusion was confirmed in all cases under radiologic assessment at 1 year postoperatively, and the bony fusion rate reached 100%. Moreover, no instrumental failure occurred during the entire follow-up period. The JOA score improved from 7.21 ± 1.62 to 12.28 ± 0.81 at the last follow-up. The ADI of 9 cases was 7.06 ± 0.85 mm preoperatively, which decreased to 2.26 ± 0.56 mm at the final follow-up. CMA improved from 103.80° ± 4.16° to 143.23° ± 7.47° postoperatively.

**Conclusion:**

With transnasal approach and lack of odontoidectomy, this method could not only treat IAAD safely and effectively, but also reduce the possibility of many complications associated with the traditional transoral approach and odontoidectomy.

## Background

The atlantoaxial joint is a complex region of the spine with unique anatomical and functional relationships [1]. Trauma, inflammation, and congenital anomalies of the odontoid can destroy the odontoid or the transverse ligament of the atlas, resulting in atlantoaxial instability. If timely treatment is not provided and the instability can be reduced by skull traction or only by posterior surgery, this condition is referred to reducible atlantoaxial dislocation (AAD), otherwise to irreducible atlantoaxial dislocation (IAAD). IAAD remains an ongoing challenge for spinal surgeons. Traditionally, transoral or transnasal odontoidectomy has been used to treat IAAD. However, these techniques carry a high risk of cerebrospinal fluid (CSF) leakage and wound infection [2]. In addition, pure odontoidectomy without posterior fixation cannot correct the swan-neck deformity and accelerates degeneration of the subaxial cervical spine [3]. Compared with the standard Endoscopic transnasal odontoidectomy, we explored alternative strategies for treating IAAD, developing a novel technique involving endoscopic transnasal anterior release and posterior reduction without odontoidectomy.

## Materials and methods

### Patients

Between September 2014 and June 2016, 9 patients (7 males, 2 females) with IAAD who underwent surgery were retrospectively analyzed. The following selection criteria were applied: patients with IAAD that could not be reduced by 2 weeks of skull traction and no bony fusion in the C1–C2 facet joints on plain radiograph and CT scan. Exclusion criteria were as follows: patients with IAAD that could be reduced by skull traction, bony fusion in the C1–C2 facet joints on plain radiograph and CT scan, and intolerance to surgery and severe heart, lung, liver, or kidney diseases. The mean age of the patients was 41.6 years, ranging from 14 to 60 years. In this study, all cases presented with occipitocervical pain, limited cervical motion, extremity numbness, weakness, and gait disturbance. The indications for surgery were neurological deficits and/or severe spinal cord compression. All patients underwent endoscopic transnasal anterior release with posterior reduction and instrumented fusion. The clinical data details of all patients are shown in Table 1.

**Table 1:** Clinical characteristics, applied surgeries, and outcomes

Case	Gender	Age (years)	Diagnosis	Surgical protocol	Reduction on radiography	Complication
1	M	39	Os odontoideum	ETAR+PRIF(C1–C2)	Complete	No
2	F	14	Os odontoideum	ETAR+PRIF(C1–C2)	Complete	No
3	M	53	Old traumatic dens fracture	ETAR+PRIF(C1–C2)	Complete	No
4	M	31	Occipitalization	ETAR+PRIF(CO–C2)	Complete	No
5	M	60	Old traumatic dens fracture	ETAR+PRIF(C1–C2)	Complete	Superficial wound infection
6	F	57	Os odontoideum	ETAR+PRIF(C1–C2)	Partial	No
7	M	40	Occipitalization	ETAR+PRIF(CO–C2)	Complete	No
8	M	45	Rheumatoid arthritis	ETAR+PRIF(C1–C2)	Complete	No
9	M	35	Old traumatic dens fracture	ETAR+PRIF(C1–C2)	Complete	No

*M* male, *F* female, *ETAR* endoscopic transnasal anterior release, *PRIF* posterior reduction and instrumented fusion

### Preoperative preparation

**Fig. 1:**
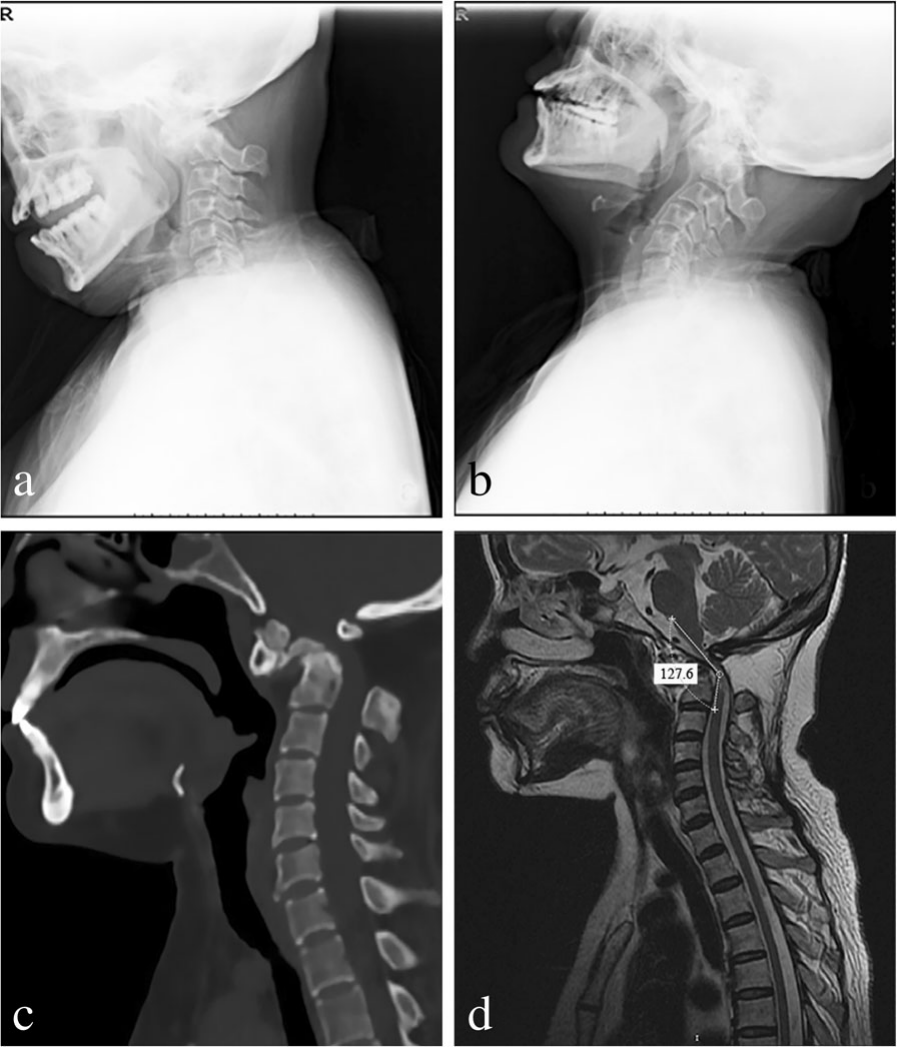
Case 6, 57-year-old female diagnosed with os odontoideum. **a**, **b** Flexion-extension radiographs showed no dynamic change of atlantodental interval (ADI) from flexion to extension. **c** Preoperative sagittal CT showing that the ADI is enlarged and the space available for the spinal cord (SAC) is decreased. **d** Sagittal MRI showed ventral compression of the spinal cord and a reduced CMA of 127.60°.

Radiological evaluations included X-ray plain film, computed tomography and 3D reconstruction (CT), and magnetic resonance imaging (MRI) (Fig. 1). The reducibility of all cases was identified by attempted skull traction under general anesthesia using fluoroscopy. The initial weight is 5 kg. After 3 min of traction, according to the result under the fluoroscopy, we gradually increase the weight (1–2 kg/time), and the maximum weight is 1/6 of the patient’s weight (10~13 kg). No “reducible” cases were observed in our study. Neurological function was monitored by intraoperative somatosensory-evoked potentials. After fiberoptic oral intubation and administration of general anesthesia, the patient was placed in the supine position with their head immobilized with a Mayfield head holder. The operating table was tilted 15° to the right to facilitate the right-handed surgeon. The patient’s nose and nares were prepared with 7.5% povidone iodine solution, followed by placement of oxymetazoline-soaked pledgets into the nasal cavity to promote vasoconstriction and decongestion of the nasal mucosa. All procedures were performed by the same senior orthopedic surgeon and the same otolaryngologist.

### Surgical procedure

#### Endoscopic transnasal anterior release

**Fig. 2:**
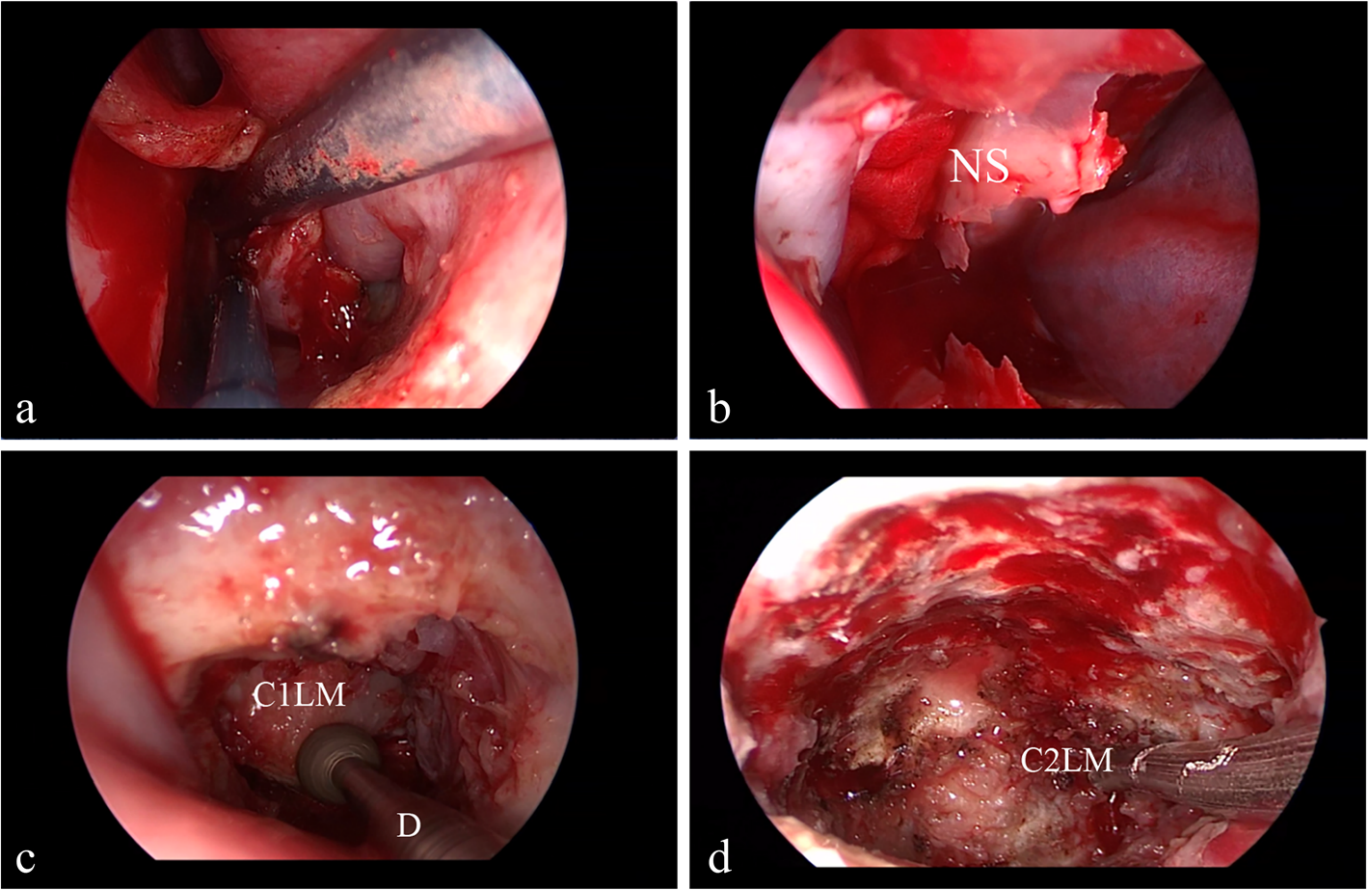
Illustration of anterior transnasal release. **a** The choana was entered and the mucosa of the rhinopharynx was dissected. **b** The posterior nasal septum was removed. **c** Expose the lateral masses of the atlas with high-speed drills. **d** Expose the lateral masses of the axis. Note: NS, nasal septum; D, drill; C1LM, lateral masses of the atlas; C2LM, lateral masses of the axis

The procedure utilized rigid-rod endoscopes that were 4 mm in diameter and 18 cm in length with lens angles of 0° and 30° mounted to a digital video camera system (Karl Storz GmbH & Co. Tuttlingen, Germany). All procedures were performed using two-nostril endoscopic techniques (Fig. 2). The bilateral middle turbinates, inferior turbinates, and the sphenoid sinus anterior wall were not resected. The bilateral inferior turbinates were lateralized, and the posterior 1 cm of the nasal septum was removed to enlarge the choana for wider exposure and to facilitate the bilateral application of instrumentations, which avoided continuously pushing the septum into the endoscope and compromising visualization. After identifying the anterior C1 tubercle via anatomical landmarks and fluoroscopy, a small linear incision was made in the midline of the nasopharyngeal mucosa. The approach is straight to the midline of the nasopharynx rather than making a U-shaped flap, decreasing the risk of damage to the adjacent structures, facilitating the wound healing process, and providing a sufficient working space. Then, the bilateral longus coli, longus capitis, and anterior longitudinal ligament were dissected caudal to the anterior ring of C1. We further dissected subperiosteally as far as the lateral margins of the C1–C2 lateral masses with high-speed drills. The anterior joint capsules, the cartilage of the bilateral C1–2 lateral joints, and any scar tissue or hyperplastic osteotylus were excised. Notably, this technique preserves the anterior arch of the atlas, and if necessary, it only involves drilling into the anterior–inferior portion around the midline while maintaining the continuity of the C1 ring. Successful release was achieved when the joint space between the lateral masses of the atlas and axis was elevated to 3–5 mm [4]. When the C1 lateral mass was levered up 3–5 mm, the C1/2 has adequate flexibility matching the aforementioned criterion, complete reduction can be achieved in most cases, and there is no need to dissect the odontoid process. Repeated elevation with resection of tissues in front of the C1 lateral mass and around the C1 anterior arch was performed until the joint space fulfilled the aforementioned criteria. After irrigation, the incision was closed. Finally, both nasal cavities were packed with expansion sponges. Note that extreme care should be taken when turning the patient to the prone position. All patients had a Philadelphia collar support to prevent atlantoaxial displacement while being turned prone. Therefore, spinal cord injury secondary to atlantoaxial instability during the transition was minimized.

#### Posterior reduction and instrumented fusion

**Fig. 3:**
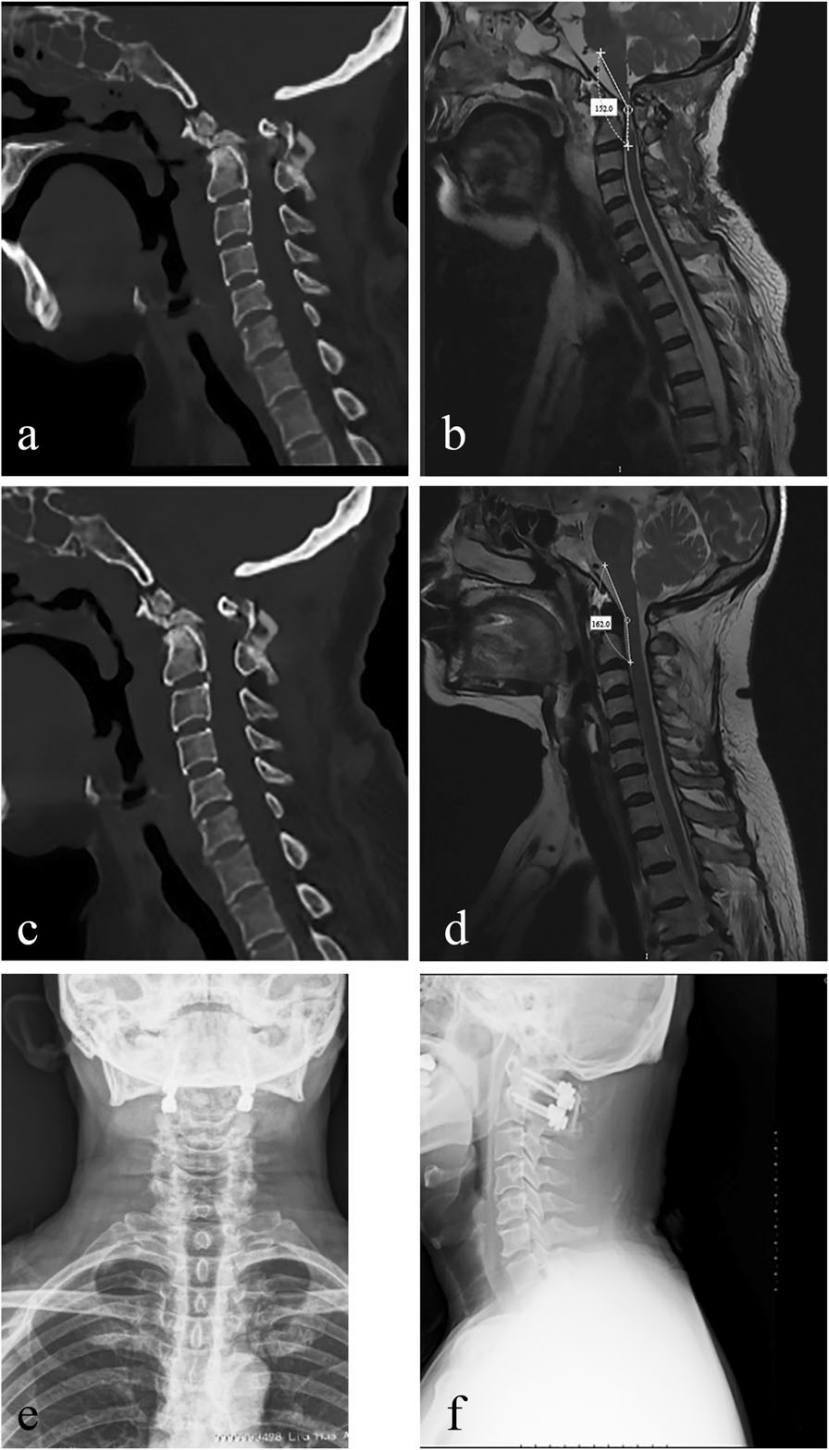
**a** Sagittal CT showed reduction and restoration 1 month after operation. **b** MRI sagittal T2-weighted films 1 month after operation showed ventral medullary decompression with CMA recovery to approximately 152.00°. **c** At 13 months follow-up, sagittal CT showed no instrument loosening, no loss of reduction, and evidence of bony fusion. **d** At 13 months follow-up, MRI sagittal T2-weighted image showed CMA recovery to approximately 162.00°. **e**, **f** Postoperative X-ray showed good internal fixation

Two different techniques were used based on the state of dislocation and bone abnormalities (Fig. 3). Occipitocervical fusion (OCF) is recommended for patients with deformities of the C1 posterior arch or lateral masses which impeded instrumentation. According to the previous study [5], we used Mimics v17.0 (Materialise, Leuven, Belgium) and 3-matic v9.0 (Materialise) to confirm whether C1–C2 pedicle screws can be fixed. During placement of the C1 pedicle screws [6], the C1 posterior arch was dissected approximately 18–20 mm (14–15 mm in children) lateral to the posterior tubercle along the posterior-inferior border subperiosteally using two Penfield dissectors. The C2 nerve root and venous plexus were dissected caudally, whereas the vertebral artery (VA) was dissected rostrally. If the height of the C1 posterior arch at the VA groove was less than 4 mm, then the “pedicle exposure technique” (PET) was performed, which we discussed in a previous study [7]. A high-speed burr was used to remove the approximately 3-mm-long outer narrow bone of the C1 posterior arch at the VA groove along the trajectory, and a 3.5-mm screw could then be inserted safely. The optimal trajectory was planned using preoperative CT scans, approximately 5 to 10° in the cephalad direction and 10 to 15° in the medial direction. In some situations, a 6 × 8 mm autologous fascia was placed between the end of the screw and the VA to protect the VA. Axial pedicle screws were inserted and connected to the C1 screws tightly with a pre-curved rod bilaterally. This technique allowed further reduction as the locking caps were tightened to the rod. As shown by the C-arm, the internal fixation was well placed and the atlantoaxial joint was sufficiently reduced (Fig. 3). After irrigation, autologous bone grafting was performed. Ultimately, the incision was closed in layers, and a drainage tube was placed inside the surgical site.

#### Postoperative management and follow-up

All patients were extubated after recovery from anesthesia and were allowed oral food intake on the next day postoperatively. The drainage tube was removed within 48 h postoperatively. The expansion sponges packed into the nasal cavities were removed on the third day postoperatively. Furthermore, all patients were required to wear a rigid cervical collar for approximately 6–8 weeks after surgery. The atlantodontoid interval (ADI) was defined as the distance from posterior edge of anterior arch to anterior edge of odontoid. The cervicomedullary angle (CMA) was defined by the angle between the two lines on the ventral side of the medulla oblongata and upper cervical spinal cord. The ADI was recorded at CT images 1 month, 1 year, and then annually after operation. Bony fusion was assessed at CT images 1 month and 1 year after operation, and this was confirmed on CT when bridging trabeculae were seen [8]. The CMA was recorded under MRI sagittal T2-weighted films 1 month after operation and the final follow-up. Complete reduction was identified as ADI ≤ 3 mm in adults and ≤ 5 mm in children, whereas partial reduction was defined as ADI of less than or equal to 5 mm but more than 3 mm in adults and 5 to 7 mm in children [4]. The Japanese Orthopedic Association (JOA) score was used to assess improvement in neurological function, and The JOA scores at 1-month and the last follow-up visit were compared with preoperative JOA scores. Redislocation and complications such as neurovascular injury, infection, or CSF leakage were recorded during the follow-up period.

### Statistical analysis

The paired *t* test was used to compare changes before and after surgery with parametric values. The *t* test was considered significant if the *P* value was less than 0.05. Statistical analyses were performed using SPSS 20.0 software.

## Results

The mean duration of the operation was 220 min (range 180–290 min), and intraoperative blood loss ranged from 240 to 810 ml (mean 390 ml). All patients were extubated after recovery from anesthesia and were allowed oral food intake on the next postoperative day. No patients required tracheostomy or reintubation. In addition, no significant complications occurred during surgery, including VA or spinal cord injuries or CSF leakage. All wounds healed without infection, except for one patient with a superficial infection related to the posterior approach, which was noted 1.5 months later and was treated successfully with intermittent debridement and antibiotics.

All patients were followed up for an average of 17 (range 13–32) months. During the follow-up, bony fusion was confirmed in all cases under radiologic assessment, 1 year after operation, and the bony fusion rate reached 100%. Eight of nine patients had complete reduction, and one had incomplete reduction based on CT scan and MRI findings at the final follow up. Moreover, no instrumental failures occurred during the entire follow-up period. All patients showed significant improvements neurologically and radiologically at the final follow-up compared with preoperative parameters (Table 2). In addition, at the final follow-up, the pediatric patient in our study experienced complete resolution of neck pain and achieved stability in various directions without the “crankshaft phenomenon” [9].

**Table 2:** Neurological and radiological outcomes

Parameters	Preoperative	One month after operation	One year after operation	Final follow-up
JOA (*n* = 9)	7.21 ± 1.62	10.11 ± 1.27^**^	12.11 ± 0.78^**^	12.28 ± 0.81^**^
ADI (*n* = 9)	7.06 ± 0.85	2.16 ± 0.43^**^	2.16 ± 0.68^**^	2.26 ± 0.56^**^
CMA (*n* = 9)	103.80 ± 4.16	137.95 ± 6.15^**^	N/A	143.23 ± 7.47^**^
		*P* = 0.00	*P* = 0.00	*P* = 0.00

^**^P < 0.01, compared with the preoperative group

## Discussion

In 1968, Greenberg first divided AAD into 2 subcategories, reducible and irreducible, and further devised a treatment strategy based on this factor as well as the etiology of the dislocation [10]. Although chronic AAD derives from various etiologies, anterior dislocation of the atlas is the most common directional endpoint. Due to progressive anterior translation, the atlas eventually loses its support from the superior C2 facets and migrates further anteriorly. The C1–C2 facets gradually reshape and the articular surface becomes increasingly vertically sloped. Then, capsules of the atlantoaxial joint, muscles, and ligaments become shortened and eventually contracted, leading to IAAD.

The most important issue to consider is whether the AAD is reducible. If preoperative dynamic X-ray confirms its reducibility, then surgical reduction and occipitocervical or atlantoaxial fusion are adequate. In our study, reducibility was investigated further under general anesthesia, and only true IAAD patients underwent anterior release and posterior reduction. Due to the presence of neck pain, muscle tension, and positional restrictions, the reducibility of AAD could not be reliably assessed with dynamic X-rays.

Currently, there is no consensus as to the ideal surgical treatment for IAAD. Historically, surgical treatment of IAAD has been performed using a pure posterior approach. One disadvantage of this technique is the need to flex the head during the operation to achieve adequate exposure of the C1–C2, increasing the risk of fatal injury to the spinal cord. Furthermore, the posterior approach may be contraindicated in cases in which the dislocated posterior arch severely compresses the spinal cord [11]. Additionally, single posterior approaches often result in incomplete reduction.

For the past several decades, the transoral approach—with or without the addition of variations such as the transmandibular–circumglossal approach or Le Fort osteotomies—has been utilized to treat symptomatic IAAD, including odontoidectomy [12, 13]. This approach provides the most direct route to the ventral craniocervical junction. However, this technique does have disadvantages. (1) The transoral approach requires opening the mouth (at least 2.5 cm) and retracting the tongue and soft palate, which can cause dental injury, edema, or necrosis of the tongue and upper airway obstruction due to edema. Therefore, the transoral approach is contraindicated in patients with micrognathia [14, 15]. In addition, palatal division can increase the risk of hypernasal speech and nasal regurgitation from velopharyngeal insufficiency (VPI). (2) Patients may need prolonged postoperative intubation or tracheostomy due to airway swelling and upper airway obstruction. Landeiro et al. reported that the rate of postoperative tracheostomy was as high as 26.3% in transoral odontoidectomy [16]. (3) The pharyngeal incision is constantly exposed to oral flora and saliva, which increases the risk of infection and the need for nasogastric tube feeding [17]. (4) The surgical area is deep and the surgical corridor is narrow under an operative microscope. Although transmandibular extension and Le Fort osteotomy can provide increased exposure, there are associated complications, including lingual nerve injury, malocclusion, mandibular pseudarthrosis, cosmetically unacceptable scarring of the lip, and the need for nasogastric tube feeding [18, 19]. Such drawbacks can increase morbidity and prolong hospitalization, prompting the search for a more minimally invasive method.

Kassamet et al. were the first to describe the successful clinical application of transnasal endoscopic odontoidectomy in a 73-year-old woman with rheumatoid arthritis and cervicomedullary compression [14]. Because the incision is made above the oropharynx and the oral cavity can be avoided without a transoral retractor or splitting of the soft palate, the endoscopic endonasal approach avoids the risk of tongue swelling and tooth damage and can improve visualization, mitigate prolonged intubation, reduce the need for enteral tube feeding, and decrease the risk of affecting phonation. Furthermore, because the wound is not constantly bathed in saliva, the risk of infection is reduced [20, 21]. Liu et al. reported that the endoscopic endonasal approach can accelerate recovery and shorten hospital stays [22]. In addition, previous studies suggested that the transnasal approach enables earlier extubation [20, 23]. In our study, extubation after recovery from anesthesia was achieved in all patients, and no tracheostomy or reintubation was needed. Moreover, patients could resume oral feeding on postoperative day 1. In a recent report of endoscopic endonasal odontoidectomy by Goldschlager et al., extubation was possible shortly after surgery, and oral feeding was resumed on postoperative day 1 on average [22]. In pediatric patients, transoral access to the craniovertebral junction is an arduous task due to the smaller mouth openings of children. Therefore, the transnasal route is an ideal alternative. Tan et al. successfully performed procedures in a patient as young as 3 years old [23]. In our study, the transnasal approach was also feasible in case 2, which was a 14-year-old child.

Intraoperative and postoperative CSF leaks were reported at relatively higher rates for transnasal endoscopic procedures (30.0% and 5.2%) than for the transoral approach (0.3% and 0.8%) [24]. CSF leaks can be caused by severe compression that causes the dura and ligaments to become very thin, improper manipulation of the last piece of the bony element of the odontoid when it is free-floating, or sharp dissection of the odontoid. Resection of the odontoid process destroys the atlantoaxial joint and the craniovertebral junction (CVJ), which inevitably affects the stability of the atlantoaxial joint. In addition, the small and deep working space makes reconstruction with bone grafts or dural tear repair with sutures difficult, predisposing the patient to a persistent CSF fistula or recurrent meningitis [17].

Spinal cord compression mainly arose from the posterior margin of a fractured C2 body and odontoid fragment and the posterior arch of the C1. The anterior tubercle of C1 and the fractured C2 body and odontoid fragment were not major factors affecting compression, so resection of the anterior tubercle and the odontoid process was not necessary [25].

After anterior release, posterior reduction as well as internal segmental fixation and fusion were often required to stabilize the atlantoaxial joint. Since subluxation can occur if patients awaited the second stage of posterior fixation and fusion, we recommend the posterior procedure at stage 1. After the posterior procedures, the atlantoaxial joints achieved reduction and fusion. Reduction is achieved sequentially by the pull strength of instruments via the posterior approach. Another advantage of the posterior approach is the realignment of the atlantoaxial joint, which can prevent subaxial degeneration due to misalignment of the cervical spine [26, 27]. In our study, only 1 patient achieved partial reduction, but this did not result in neurological deficits. Partial reduction can be caused by the lack of intra-spinal canal manipulation with the posterior approach; therefore, soft tissue and scar tissue may remain within the spinal canal and impede the reduction of C1 to its proper anatomical position. In terms of the instruments for posterior fixation, we recommend C1–C2 pedicle screws because they provide more pullout strength, less irritation of the C2 nerve root and venous plexus, and a more visible entry point [28, 29]. For pediatric patients, many studies have verified the feasibility of placing C1 pedicle screws, even if the height of the C1 posterior arch is < 4 mm [7, 30]. In our study, all patients achieved bony fusion, as determined by radiography, and no implant failures or migrations were observed radiographically at the last follow-up.

The limitations of the study are as follows: low number of subjects, narrow operative field, steep learning curve, and limit in the caudal direction to the inferior base of C2.

## Conclusion

With transnasal approach and lack of odontoidectomy, this method could not only treat IAAD safely and effectively, but also reduce the possibility of many complications associated with the traditional transoral approach and odontoidectomy.

## Abbreviations

IAAD: Irreducible atlantoaxial dislocation
CSF: Cerebrospinal fluid
CT: Computed tomography
MRI: Magnetic resonance imaging
OCF: Occipitocervical fusion
VA: Vertebral artery
PET: Pedicle exposure technique
ADI: Atlantodontoid interval
CMA: Cervicomedullary angle
JOA: Japanese Orthopedic Association
